# Supplementary material for: Local orthorhombic lattice distortions in the paramagnetic tetragonal phase of superconducting NaFe1−xNixAs
Source: Nat Commun. 2018 Aug 7;9:3128. doi: 10.1038/s41467-018-05529-2 (PMC6081486; doi:10.1038/s41467-018-05529-2)
Supplement: Supplementary file 1 — Supplementary Information [file 41467_2018_5529_MOESM1_ESM.pdf]

Supplemental Information: Local orthorhombic lattice distortions in the paramagnetic tetragonal phase of superconducting $NaFe_{1-x}Ni_xAs$

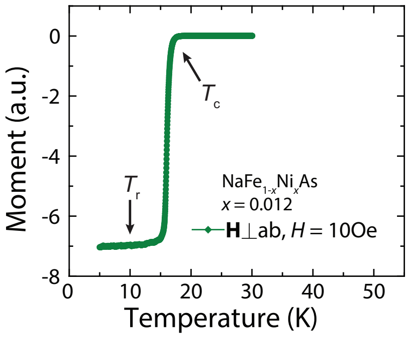

Supplementary Fig. 1: Temperature dependence of the magnetic susceptibility for $NaFe_{1-x}Ni_xAs$ ($x = 0.012$). T_c and T_r marked by arrows are superconducting transition temperature and temperature when static antiferromagnetic order is suppressed, respectively.

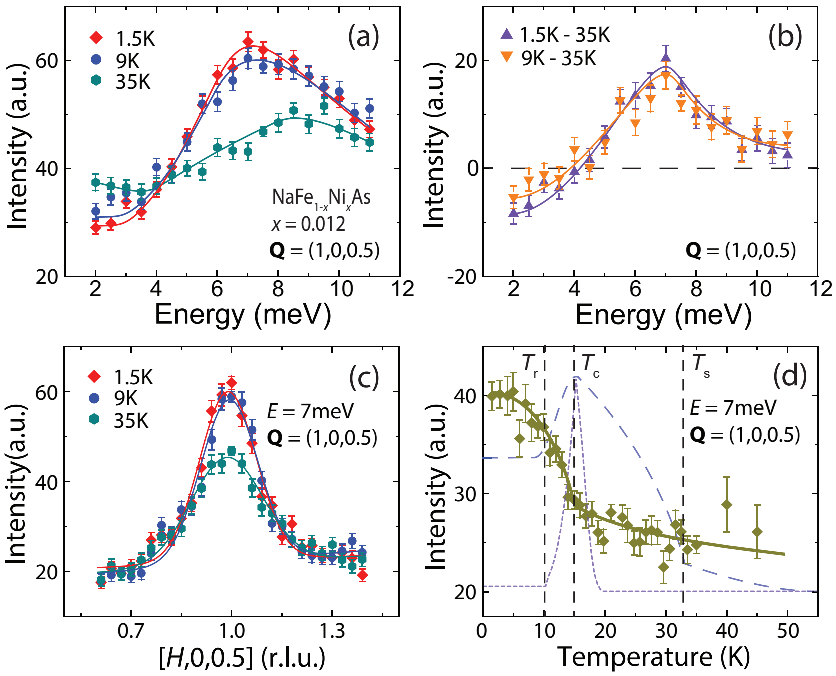

Supplementary Fig. 2: Inelastic neutron scattering measurements for $NaFe_{1-x}Ni_xAs$ ($x = 0.012$). (a) Energy scans at $\mathbf{Q} = (1, 0, 0.5)$ for $NaFe_{1-x}Ni_xAs$ with $x = 0.012$ at $T = 1.5$, 9 and 35 K. (b) Energy scans at $T = 1.5$ and 9 K in (a) after subtracting the scan at 35 K. (c) Constant-energy scan along $[H, 0, 0.5]$ with $E = 7$ meV at $T = 1.5$, 9 and 35 K. (d) Temperature dependence of magnetic excitations at $E = 7$ meV and $\mathbf{Q} = (1, 0, 0.5)$ in $NaFe_{1-x}Ni_xAs$ with $x = 0.012$. The magnetic and structural order parameters from Fig. 2(h) and 3(b) are over-plotted for comparison. All vertical error bars in the figure represent statistical errors of 1 s.d.

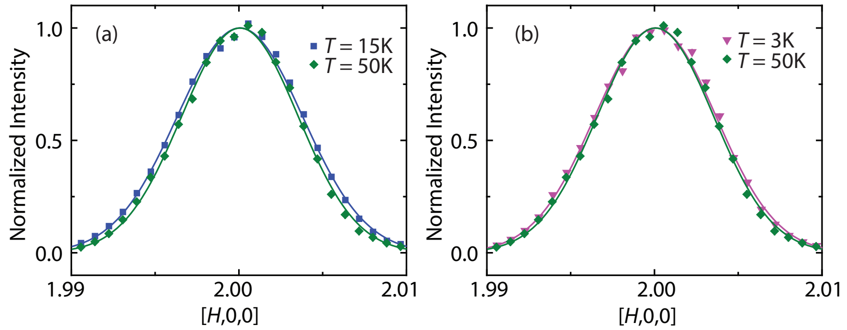

Supplementary Fig. 3: Structural measurements for $NaFe_{1-x}Ni_xAs$ ($x = 0.012$) using SPINS. (a) Scans of the structural Bragg peak $\mathbf{Q} = (2, 0, 0)$ along the H direction for $NaFe_{1-x}Ni_xAs$ with $x = 0.012$ are compared between 15 K and 50 K. Similarly, a comparison between 3 K and 50 K is shown in (b).

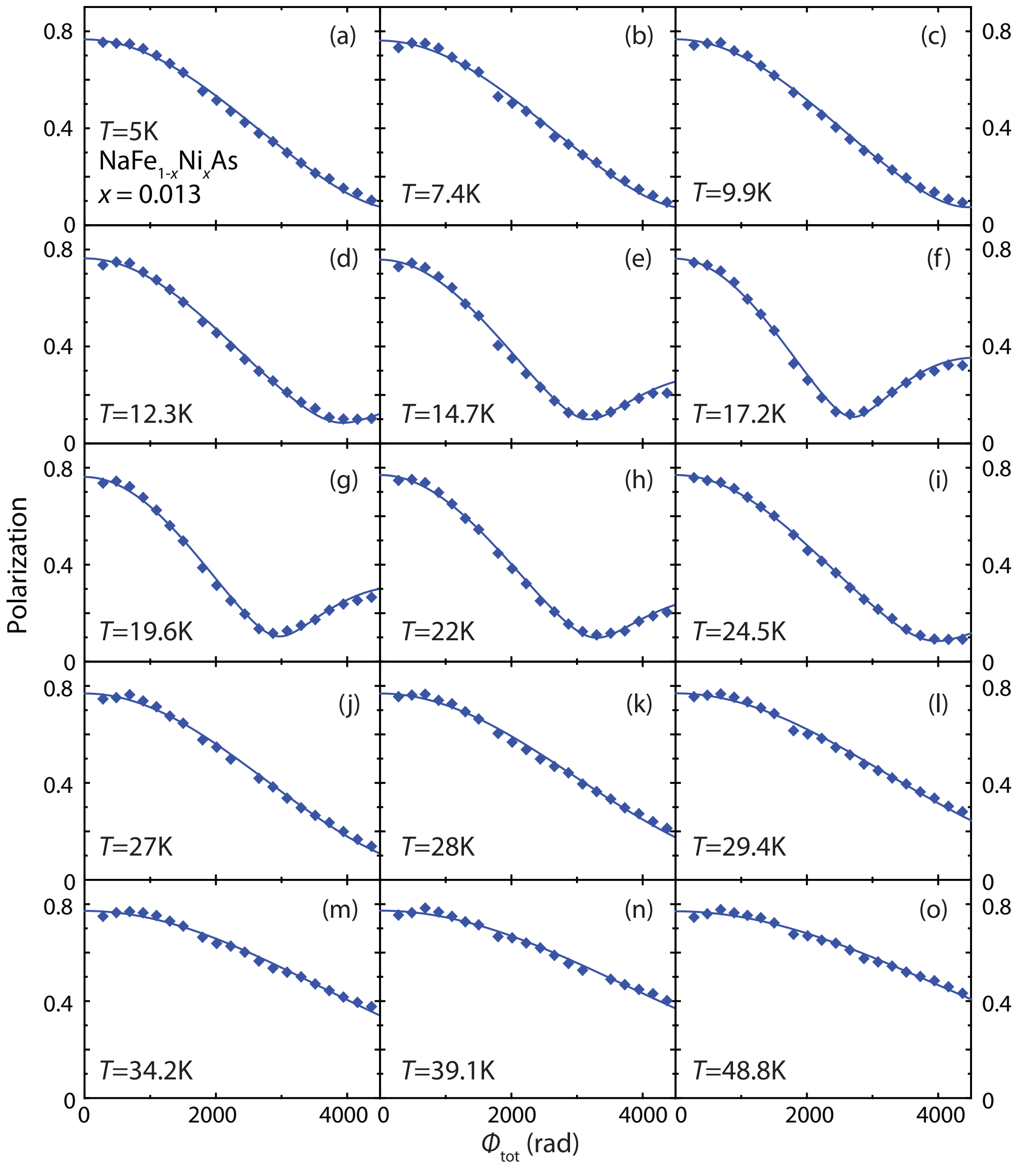

Supplementary Fig. 4: Neutron Larmor diffraction measurements of $P\phi_{\rm tot}$ at $\mathbf{Q} = (4, 0, 0)$ for $NaFe_{1-x}Ni_xAs$ ($x = 0.013$) at different temperatures. Solid lines are fits to Eq. 3 in Methods section of the main text.

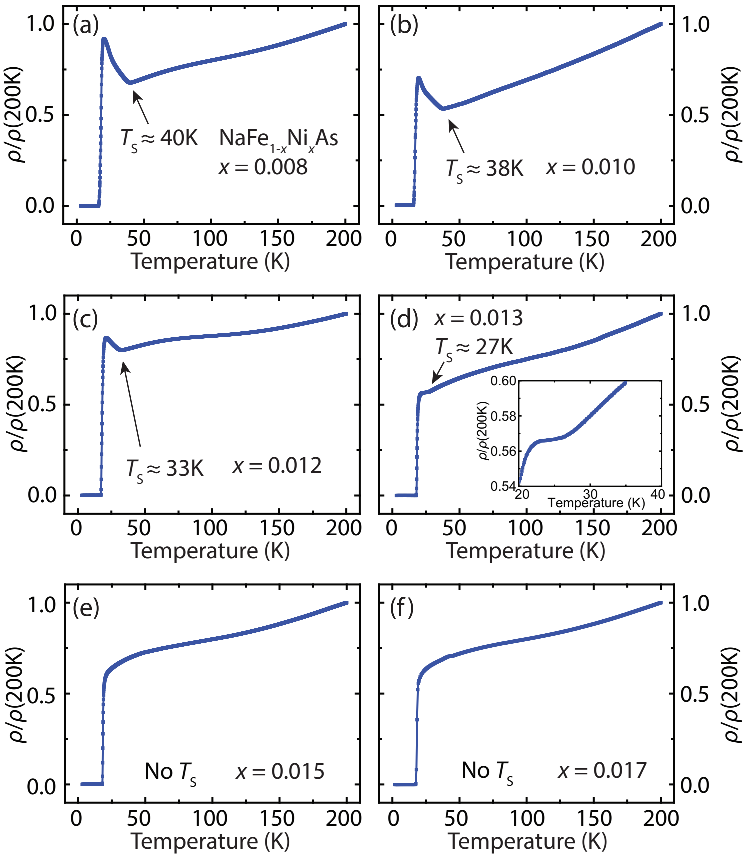

Supplementary Fig. 5: Temperature dependence of in-plane resistivity for $NaFe_{1-x}Ni_xAs$ single crystals. The inset in (d) shows a zoom-in to highlight the weak kink associated with the structural transition T_s in the $x = 0.013$ sample.
